# Giving voice to the next generation in paediatric cardiology and congenital heart diseases

**DOI:** 10.1093/ehjcr/ytag624

**Published:** 2026-08-26

**Authors:** Sara Moscatelli, Ruth Heying, Ina Michel-Behnke, Colin J McMahon, Sylvia Krupickova, David Duncker, Philipp Sommer

**Affiliations:** Centre for Paediatric Inherited and Rare Cardiovascular Disease, Institute of Cardiovascular Sciences, University College London, London WC1N 1DZ, United Kingdom; Centre for Inherited Cardiovascular Diseases, Great Ormond Street Hospital, London WC1N 3JH, United Kingdom; Department of Cardiovascular Developmental Biology, Pediatric Cardiology, University Hospitals Leuven, KU Leuven, 3000 Leuven (UZ Leuven), Belgium; Hospital of Children and Adolescent Medicine, Helios Dr. Horst Schmidt Kliniken, Wiesbaden 65199, Germany; Department of Paediatric Cardiology, Children’s Health Ireland, Crumlin, Dublin, D12 N512 (CHI), Ireland; Cardiac MRI Department, Royal Brompton Hospital, Guy's and St Thomas’ NHS Foundation Trust, Sydney St, London SW3 6NP, UK; Paediatric Cardiology Department, Royal Brompton Hospital, Guy's and St Thomas’ NHS Foundation Trust, Sydney St, London SW3 6NP, UK; Imperial College London, Exhibition Road, South Kensington, London SW7 2AZ, UK; Hannover Heart Rhythm Center, Department of Cardiology and Angiology, Hannover Medical School, Hannover, 30625, Germany; Clinic for Electrophysiology, Herz- und Diabeteszentrum NRW, Georgstr. 11, Bad Oeynhausen 32545, Germany

**This editorial refers to ‘Percutaneous management of atrial flow regulator thrombosis in failing Fontan: a case report’, by M. Quarta et al. doi:https://doi.org/10.1093/ehjcr/ytag604**

Despite the remarkable advances in cardiovascular medicine, paediatric cardiology remains a field with comparatively limited academic visibility. This is not due to a lack of scientific or clinical complexity but rather reflects structural challenges inherent to the specialty. Smaller patient populations, fewer large-scale studies, and consequently a more limited number of dedicated high-impact journals are all factors contributing to reduced opportunities for dissemination of research and clinical experience.

For early-career clinicians and researchers, these barriers are particularly evident. Young professionals in paediatric and congenital cardiology often face difficulties in publishing their work, not necessarily due to lack of quality, but because of restricted access to appropriate platforms.^1^ In many cases, valuable clinical insights—especially those derived from single cases or small series—remain under-recognised despite the well-established educational value of case reports.^2^

From this perspective, we developed the Young Community Clinical Case Competition, in collaboration with the Association for European Paediatric and Congenital Cardiology (AEPC) Educational Committee plus Junior members and *EHJ Case Reports*.

Our aim was simple but ambitious: to create a dedicated space for young clinicians to share their work, engage with an international audience, and access opportunities for publication that might not otherwise be readily available within their local institutions. At its core, the initiative sought to address a broader gap—not only in publication opportunities, but also in visibility, confidence, and academic engagement.

Case-based learning remains central to paediatric cardiology. The diversity of congenital and inherited conditions, often encountered in small numbers, makes individual clinical cases particularly valuable for collective learning. Yet, these same characteristics can make such work more difficult to publish in traditional research-focused journals. By focusing on clinical cases, this competition aligns naturally with both the realities of the field and the educational needs of its community.

A distinctive feature of this initiative was its interactive format. Participants were not only invited to submit their cases, but five finalists were also selected to present them during a dedicated session within a regular webinar series (‘AEPC Heart Beats for Education’), developed specifically for fellows. This created an environment that extended beyond passive dissemination, encouraging discussion, critical appraisal, and peer learning. For many young clinicians, this represented a rare opportunity to present their work at an international level, fostering both scientific communication skills and professional confidence.

Importantly, the competition also reflects the broader mission of the AEPC, and in particular its Educational Committee, in promoting education and inclusivity within paediatric cardiology. By providing a structured yet accessible platform, the initiative helps to reduce disparities related to institutional resources and geographical differences. Not all trainees have equal access to mentorship, research infrastructure, or publication pathways; initiatives such as this can help to bridge that gap.^3^

The collaboration with *EHJ Case Reports* has been instrumental in this process. As a journal with a strong educational focus and an established role in disseminating clinically relevant cases, it represents an ideal partner for such an initiative.^4^ The possibility of publication offers a tangible incentive while also ensuring that high-quality cases contribute to the wider scientific community.

Beyond the immediate outcomes, the value of this competition lies in its broader impact. It encourages young clinicians to engage actively with academic medicine, to reflect on their clinical practice, and to recognize the importance of sharing knowledge. It also fosters a sense of belonging within an international network, which is particularly important in a highly specialized field such as paediatric cardiology.^5^

From our perspective as organizers, the response to this initiative has been both encouraging and inspiring. The quality of submissions, the enthusiasm of participants, and the level of engagement during presentations all highlight the need for such platforms. More importantly, they demonstrate that young clinicians are eager to contribute to the scientific community when provided the opportunity to do so.

Looking forward, initiatives like the Young Community Clinical Case Competition should not be viewed as isolated events but as part of a broader effort to strengthen academic pathways in paediatric cardiology. Given the great success of this first edition, a second edition of the competition will be held, with the call for submissions opening in September 2025; the initiative will follow the same structure and interactive format, including case submission, selection of finalists, presentation within the AEPC Heart Beats for Education webinar series, and the opportunity for selected cases to be considered for publication. Expanding opportunities for presentation and publication, fostering mentorship, and promoting inclusive participation will be essential to support the next generation.

In conclusion, this competition represents more than an educational exercise—it is a step towards addressing structural challenges within the field. By creating space for young professionals to be seen and heard, we contribute not only to their individual development, but also to the collective advancement of paediatric cardiology.

The five finalists who presented their cases were

- Maria Teresa Quarta (Italy), with a case on percutaneous management of Atrial Flow Regulator thrombosis in a failing Fontan;
- Andrea Greco (Spain), who presented a case of severe neonatal long QT syndrome type 3 due to a *de novo* SCN5A inactivation-gate variant, managed with genotype-directed therapy combined with early LCSD;
- Manuela Lopes (Portugal), with a case of left pulmonary artery arising from the common carotid artery in DiGeorge syndrome, managed with ductal stenting as a bridge to repair;
- Jacob Gilchrist (United Kingdom), who described successful percutaneous intervention for post-operative coronary artery stenosis in a child; and
- Roberta Di Cosola (Italy), who presented a case of left ventricular dysfunction and massive intracavitary thrombus in unrepaired tetralogy of Fallot with endomyocardial fibroelastosis.

Among these, the cases presented by Andrea Greco, Maria Teresa Quarta, and Jacob Gilchrist were selected as winners, reflecting both scientific merit and clinical relevance. This further emphasizes the inclusive and developmental nature of the initiative, ensuring that high-quality contributions are recognized and disseminated even beyond the competition outcomes.

**Figure 1:**
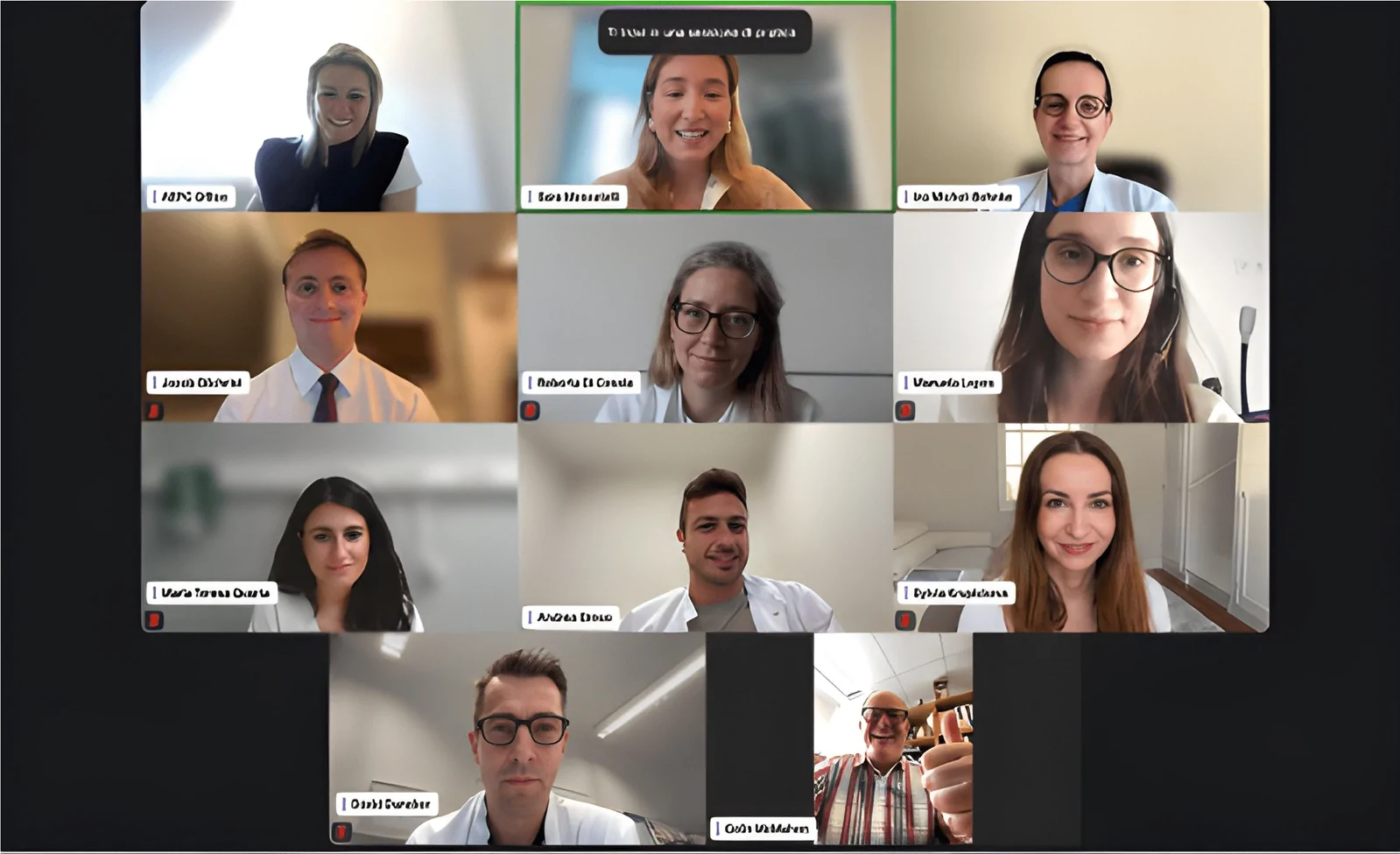
Group photograph of the jury and finalists at the beginning of the young community clinical case competition, held virtually via the AEPC zoom platform. The image includes members of the jury: Prof. David Duncker (Executive director, EHJ Case Reports), Dr Silvia Krupičková (EHJ Case Reports Editorial Board Member and AEPC member), Dr Ruth Heying (Chair of the AEPC Educational Committee), Dr Colin McMahon (AEPC Treasurer and Educational Committee member), and Prof. Ina Michel-Behnke (AEPC Secretary General and Educational Committee member). Also pictured are representatives from the AEPC Office, including Linda Bosschers, and Dr Sara Moscatelli (AEPC Junior Councillor 2024–2027; Research Fellow at the Institute of Cardiovascular Science, UCL, and Great Ormond Street Hospital, London, UK), promoter and coordinator of the initiative, together with the finalists of the competition.

## Lead author biography

Dr Sara Moscatelli is a paediatric cardiologist fellow at Great Ormond Street Hospital and a PhD candidate at University College London. Her clinical and research interests focus on inherited cardiovascular conditions in children and adolescents, particularly arrhythmogenic cardiomyopathies. She is actively involved in scientific publishing, medical education, and international collaborations and serves as Junior Councillor and Educational Committee member of the Association for European Paediatric and Congenital Cardiology (AEPC). She is committed to supporting early-career professionals and promoting women's leadership in cardiology.
